# UK parents' help‐seeking for child sleep: A qualitative investigation into parental preferences and reservations about resources

**DOI:** 10.1111/hsc.13959

**Published:** 2022-08-09

**Authors:** Georgia Cook, Jane V. Appleton, Luci Wiggs

**Affiliations:** ^1^ Centre for Psychological Research Oxford Brookes University Oxford UK; ^2^ Formerly OxINMAHR (Oxford Institute of Nursing Midwifery and Allied Health Research), Faculty of Health and Life Sciences, Oxford Brookes University Oxford UK (retired)

**Keywords:** child sleep, help‐seeking, preferences, reservations, resources

## Abstract

Child sleep is a common parental concern and there is an array of resources available to parents. However, an exploration of UK parents' help‐seeking behaviours around child sleep is lacking. This study sought to identify the resources parents use to seek information and help for child sleep, as well as to explore what factors parents prefer about certain sources and their reservations about using other resources. Parents of 6‐36 month old children residing in the United Kingdom (UK) completed an online questionnaire between October 2015 and October 2016 about their use, opinions and experiences regarding resources for child sleep. Quantitative data were descriptively analysed and thematic analysis was conducted on parents' open‐ended text responses. Participants were 266 UK parents (97% mothers). Parents' ages ranged from 21 to 45 years (*M* = 33.49 years, *SD* = 4.71) and all resided in the United Kingdom (UK). General Internet searches were the most commonly reported source used by 47% of parents with a range of other informal resources also frequently consulted. Health Visitors (HVs) were the most accessed healthcare professional reportedly consulted by 38% of parents. Seven themes represented parental preferences for their resource use. Most strongly endorsed included a desire for information from other parents, particularly those with practical experience and accessing information that aligned with their parenting values. Parents preferred sources that provided support and reassurance, as well as those that afforded parents the ability to select relevant elements from a range of information. Seven themes represented parents' reservations about resources. Most strongly endorsed were concerns about reliability, being judged and challenges associated with filtering vast amounts of information. Parents reported having reservations towards sources if they had a previous negative experience with the source. Possible implications of the findings and specific suggestions about how existing and future resources could be adapted to better meet parents' needs are highlighted.

**What is known about the topic**

- Very little is known about parental help‐seeking behaviours for child sleep generally, but even less about parents in the UK
- It is not known what preferences parents have about resources for child sleep nor what can cause parents to have reservations about resources when seeking help for child sleep

**What does this study add**

- Identifies the resources parents report accessing for information, advice and help for child sleep
- Highlights parentally reported preferences and reservations about the nature and content of resources for child sleep
- Provides suggestions as to how current and future sources could be adapted to better meet parents' needs

## 1 INTRODUCTION

Child sleep and child sleeplessness problems (CSPs) are common worries for parents (Mindell et al., 2015; Porter & Ispa, 2013; Trajanovska et al., 2010) affecting up to 25% of families (Sadeh et al., 2009; Wake et al., 2006). Whilst up‐to‐date United Kingdom (UK) prevalence data is lacking, just under half (46.3%) of 266 parents of infants and toddlers in a recent UK sample reported their child's sleep to be problematic to some extent (Cook, 2018). In many cases, these problems are persistent (Byars et al., 2012; Williamson et al., 2019). This is of note, given that CSPs can be associated with adverse outcomes for the functioning and well‐being of both the child (Magee & Hale, 2012; Scher et al., 2010; Sivertsen et al., 2015; Spruyt et al., 2008) and family (Bayer et al., 2007; Martin et al., 2007). Parents appear particularly concerned with aspects of a child crying associated with sleep, naps, and night wakings (Simard & Pilon, 2021). There are multiple informal and formal healthcare resources available to parents that offer information, advice and/or help for child sleep. Informal sources include other parents, friends, family members, general Internet searches or online forums, children and parenting groups and some books. Formal healthcare resources tend to include a range of healthcare professionals (HCPs), such as doctors, paediatricians, health visitors (HVs), midwives and practice nurses amongst others, as well as some printed literature and websites (Brady & Guerin, 2010; France et al., 2003; Henderson et al., 2013; Porter & Ispa, 2013; Simard & Pilon, 2021; Stremler et al., 2013; Tsai et al., 2014). HV is a term which, in the UK, represents a registered nurse or midwife who has undertaken additional training in community public health nursing; the focus of the role is to work with families with children up to 5 years of age.

There has been limited exploration of parents' help‐seeking behaviours in relation to child sleep. From previous research, it appears there is geographic (and possible cross‐cultural) variation in how and from where parents seek help for child sleep. For example, mothers in Taiwan, Canada and the United States of America, prefer to use informal sources (Johnson, 1991; Stremler et al., 2013; Tsai et al., 2014) whilst mothers from New Zealand and Australia prefer to seek help from formal healthcare resources (Henderson et al., 2013; Trajanovska et al., 2010). Canadian mothers have also reported being satisfied with formal healthcare professional consultations (Simard & Pilon, 2021). A UK‐centric study of parents of 3‐12 year old children suggests that what is important to parents is that information comes from those with relevant practical experience, with parents being accepting of this type of information coming from informal sources such as family and friends (Hatton & Gardani, 2018). Given the differences in healthcare systems across countries, it is perhaps unsurprising that there is geographic variation, and it is unlikely results from different countries can be generalised.

Previous research has predominantly focused on parental use of formal healthcare resources (Blunden et al., 2004; Cook et al., 2020; Hsu et al., 2017; Morrell, 1999), yet parents may also use other resources and so this emphasis may underrepresent the proportion of parents who seek help and how they approach doing so. Only a few studies have explored help‐seeking in a UK sample (Cook et al., 2020; Morrell, 1999). For example, Morrell (1999) explored advice seeking in mothers of 13–16 month olds. Findings suggested just over 40% of mothers who perceived their child had a CSP and/or mothers whose children met a research definition of a CSP had sought help or advice for child sleep. However, only help‐seeking from formal healthcare resources, such as doctors or HVs, was explored and it remains unclear why some mothers who perceived their child to have a CSP had not sought professional help or if mothers had made use of sources other than HCPs. Recent studies have suggested that some parents have concerns about using HCPs for advice or support around child sleep due to a perceived lack of HCPs' knowledge or training (Cook et al., 2020; Hatton & Gardani, 2018). There remains a lack of evidence from the UK regarding what resources parents use and what factors encourage or cause parents to have reservations about the use of resources for child sleep.

A small number of studies have explored variables that affect parental use of sources for child sleep. Maternal age (under 25 years), socioeconomic status (SES, lower and mid) (Henderson et al., 2013) and information which is not aligned with preferred parenting style or beliefs (Tsai et al., 2014) have been found to negatively influence parental help‐seeking for child sleep. In the UK, for most common CSPs, first‐line treatments recommended by HCPs would be based on evidence‐based, behavioural methods (Meltzer & Mindell, 2014; Mindell et al., 2006). However, not all parents may want or be capable of successfully implementing these types of sleep interventions (Blunden et al., 2011; Cook et al., 2020; Tse & Hall, 2008). Yet, it is uncertain what aspects of the sources themselves encourage or discourage parents from using certain resources for child sleep.

Therefore, even though a wide range of resources are available to parents for information, advice or help for their child's sleep little is known about what sources parents use and why. The current study sought to identify the resources parents use to seek information and help for child sleep, as well as to explore what factors parents prefer about certain sources and their reservations for using other resources. It is hoped these results could contribute to ensuring the provisions available for child sleep are informed by what parents want and would best meet their needs.

## 2 MATERIALS AND METHOD

### 2.1 Participants and recruitment

Details of the research project were displayed via an advert with an embedded online questionnaire link on (i) social media and (ii) online parenting websites alongside various national and local parenting groups. In addition, (iii) emails including the background to the study and the study advert were sent to parents of children in the study age range via the University's Babylab (active child research group within the University, which has an extensive database of parents in the local area who are interested in participating in child research) and (iv) Individuals/groups who had an interest in the research topic (primarily sleep consultants and experts) also disseminated an advert for the project. The final sample of parents completed the questionnaire between October 2015 and October 2016. Recruitment was from social media sites (Facebook, 58.6% and Twitter, 10.9%), ‘other’ (7.5%), word of mouth (7.1%), University Babylab (6.0%), parenting group (5.3%), and online advert (3.4%).

Participants were 266 UK parents (97% mothers) of 6–36 month olds. Parents' ages ranged from 21 to 45 years (*M* = 33.49 years, *SD* = 4.71, from age data given by 261 parents). This was a convenience sample, with participants completing the questionnaire via adverts disseminated online. To be eligible for the study parents needed to have a child aged 6–36 months, be living in the UK and have sufficient English language skills to understand and complete the online questionnaire.

### 2.2 Measures

#### 2.2.1 Parental help‐seeking questionnaire development

As there was no existing tool to assess parental help‐seeking behaviours for child sleep, a questionnaire was designed for this study. Researchers generated questionnaire items (*n* = 29) that addressed topics pertinent to the project. In collaboration with clinical and research sleep specialists, items were refined, predominantly to improve clarity. The final questionnaire consisted of 24 items and was piloted with two sets of parents. Based on feedback, supplementary open‐ended response options were added to allow parents to describe their own experiences and opinions in addition to choosing pre‐selected options.

#### 2.2.2 Final questionnaire

**FIGURE 1:**
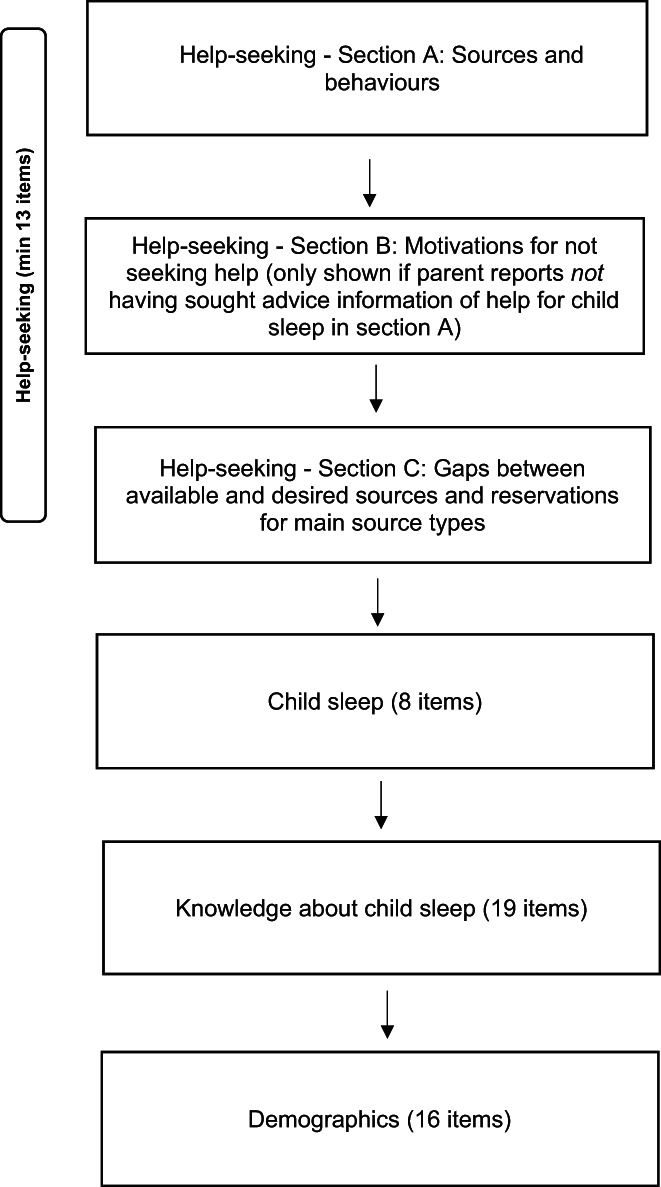
An illustration of survey content and flow

Each participant completed an online questionnaire about their help‐seeking behaviours, child sleep, knowledge about child sleep and familial demographics. Sleep was assessed using the Brief Infant Sleep Questionnaire (BISQ) (Sadeh, 2004) and knowledge about child sleep using the Sleep Practices and Attitudes Questionnaire (SPAQ‐K) (adapted from Grandner et al., 2014) although these variables are not reported in the current paper. See Figure 1 for an illustration of the survey flow. Results in the current paper focus on parents' responses to questions in section A which assessed: (i) What resources parents had used for advice, information or help for child sleep addressed by the question ‘From where have you sought advice, information or help for your child's sleep?’ This question had 14 possible response choice options, from which parents were asked to select all that applied (see appendix A for full list); (ii) Why were those the most useful sources for them addressed by the question, ‘From those you have selected above what were the most useful to you for advice, information or help for your child's sleep and why?’. Parents were provided with an open text box in which to type their response and were asked to provide as much detail as possible and from Section B, which assessed; (iii) Any reservations or factors that act as barriers to using key resource groups (health professional, internet, family or friends, written information or other) via the question ‘If you have any reservations about using any of these options for advice about your child's sleep now or in the future what are they?’. Again, parents were provided with an open text box for their response.

### 2.3 Analysis method and presentation

Quantitative (multiple choice) data is descriptively presented. Thematic analysis was conducted on open text box responses, in‐line with Braun and Clarke's (2006) methodology. This process included: (1) Data familiarisation achieved through reading and re‐reading transcripts; (2) Initial coding which involved adding codes to salient aspects of the transcript relevant to the research question; (3) Theme identification, which was achieved through the grouping of relevant associated codes into initial themes; (4) Theme review which involved reflecting on the initial themes and that they accurately represented the coding and data set, making amendments to themes (collapsing or splitting themes where necessary); (5) Theme refinement and naming which required constructing or finalising theme names and producing a clear description of what the theme represented; and finally (6); Writing up the analysis in full including integrating the narrative account and participant quotes.

This analysis method was chosen due to its theoretical flexibility, which was appropriate for the exploratory nature of the study. Analysis was conducted from an inductive perspective where participants' words formed the foundation from which themes were generated. To ensure the credibility of the analysis process two supervisors (LW and JA) and a qualitative researcher (KH) not involved in the project were extensively involved in the reviewing, discussion and refinement of codes and themes (independently reviewing over 10% each of the total data set). Themes are presented concurrently with descriptions of how each theme was conceptualised by the researchers alongside supporting quotations from participants. In a small number of places, obvious typographic or grammatical errors in quotations were corrected for presentation.

### 2.4 Procedure

Ethical approval was issued by the University's Research Ethics Committee (study number 150932). The questionnaire was accessed via an anonymous weblink, which was included in the adverts that were distributed by various means (as reported in the recruitment section). When a participant clicked on the weblink they were initially presented with the information sheet, on the same page participants also provided informed consent by ticking a box. Participants were then able to access the questionnaire (hosted by Qualtrics). Participants were able to cease completing the questionnaire at any time by exiting their web browser. Analysis was only conducted on fully completed and submitted questionnaires. All submitted responses, which included a valid email address, were included in a prize draw (£50 Amazon voucher). The questionnaire was open between October 2015–October 2016 and during this time, adverts and links were regularly re‐posted and updated.

## 3 RESULTS

### 3.1 Participant characteristics

Full sample demographics are reported elsewhere (Cook et al., 2020). In summary, over three‐quarters of the sample self‐identified as being white British (87.2%, based on 262 reported ethnicities) and well‐educated with 76.3% (based on 259 reported education levels) reporting that they held either a university degree or had completed further postgraduate education. Most of the sample (79.7%, based on 265 reported occupations) was employed and 19.5% reported being full‐time parents.

Two hundred and sixty‐five participants reported their living location: town (43.6%), village (26.3), city (21.8%) or rural (7.9%) location. The most common living arrangement (based on 264 participants' reporting) was with a partner and child/ren (91.7%). Responses came from many of the UK counties (*n* = 48).

Respondents were parents of 139 (52%) boys and 127 (48%) girls. Inclusion criteria stated that children had to be aged 6–36 months; the children had a mean age of 19.41 months (*SD* = 9.26).

### 3.2 Parents' use of resources

**FIGURE 2:**
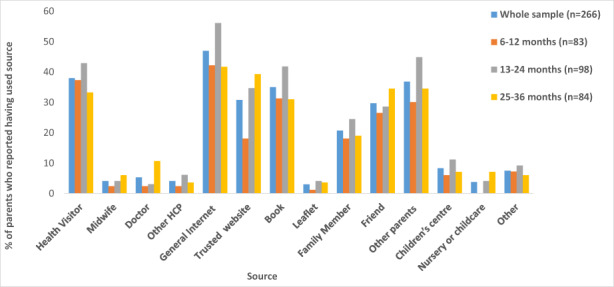
The percentage of parents who reported having utilised different sources to obtain advice, information, or help for their child′s sleep across the whole sample (6–36 months) and specific age groups; 6–12 months, 13–24 months, and 25–36 months.

A considerable proportion of parents (*n* = 179, 67.3%) reported having sought advice, information, or help for their child's sleep at some point. The proportion of parents who reported having used individual source types for the sample as a whole and in age bandings are displayed in Figure 2. Across the whole sample and all age groups, general Internet searches were the most common source of information, reportedly having been used by 47% of the whole sample. Health Visitors (HVs) were the most commonly used formal healthcare resource, reportedly having been used by 38% of the whole sample. Other parents, books, and trusted parenting or health websites were also commonly reported used resources.

### 3.3 Parental preferences and reservations

Thematic analysis of parents' open‐ended text box responses revealed themes, which cut across individual source types and represented overarching reasons why parents held preferences or had reservations about resources. Four of the most commonly reported pertinent themes for each question (preferences and reservations) are presented in full below. Three additional themes, descriptions and illustrative quotations are presented in Tables 1 (preferences) and 2 (reservations).

**TABLE 1:** Reasons parents found sources useful for child sleep

Themes	Description	Example quotations
A. Experience	Source or information is based on relevant, personal or professional experience. Information that incorporated real‐life practicalities was valued	*[online group] ‘They were able to share real experiences and give bespoke*, *appropriate advice’ (Mum*, *31 of boy 15 months)* *‘It's useful to be able to read different people's experiences to see what worked for them’ (Mum*, *31*, *of girl 35 months)* *[Friends or other parents] ‘…who are going through / have been through a similar thing. Empathy and experience are a great thing!’ (Mum*, *35*, *of boy 19 months)* *‘Having practical advice is invaluable when you are a new parent. I want advice from other parents*, *who know the struggle*, *can relate*, *and can give practical advice’ (Mum*, *29*, *of boy 17 months)*
B. Sources that were in line with the parenting approach	The source or its content was in line with the parent's desired parenting style or approach to parenting. This included actively using or avoiding sources due to its inclusion or avoidance of certain approaches.	*‘They provide support and advice that is child centred and avoids any “sleep training” methods’ (Mum*, *34*, *of boy 25 months)* *‘Friends and peers with similar values to me’ (Mum*, *30*, *of girl 6 months)* *‘Any book that advocates CIO or CC (Cry It Out and Controlled Crying) should be avoided’ (Mum*, *33*, *of boy 27 months)* *‘I wanted gentle/attachment parenting advice’ (Mum*, *31*, *of boy 15 months)*
C. Support and re‐assurance	Source provided support and reassurance, both in relation to child sleep generally, as well as specific sleep issues	*‘I've found gentle parenting websites and social media groups have a similar viewpoints to me so I've found these the most useful in reassurance and support’ (Mum*, *32*, *of girl 20 months)* *‘Although friends are only really able to share their own anecdotal experiences these can feel far more relatable than statistics and their reassurances have been invaluable to me’ (Mum*, *36*, *of girl 17 months)* *‘…[I] prefer to read what people suggest / experiences online and speak to people who I know well and trust’ (Mum*, *35*, *of girl 35 months)*
D. Having access to a broad range of information and the ability to select what is relevant	Parents desired having access to a broad range of information (which may come from various sources) and included a range of diverse options and ideas, from which they were able to select only aspects that they agreed with, or felt would be useful in their individual circumstances	*‘All of them [were useful] in different ways. Taking the advice from each that I thought was relevant to us’ (Mum*, *30*, *of girl 25 months)* *‘I find it helpful to get lots of info and decide what works best for me. I would prefer to get a wide range of thoughts and the be able to try different things at my own pace’ (Mum*, *38*, *of boy 33 months)*
E. Normal child sleep information or normalised child sleep behaviour	Source informed parents about normal child sleep or normalised child sleep behaviour. Many parents sought to obtain information which allowed them to make a comparison between their own child with children of a similar age	*‘All the resources above have been helpful as they have normalised my baby's sleep pattern which has prevented me from problematising him…’ (Mum*, *37*, *of boy 8 months)* *‘…helping me to understand sleep and reasons why baby is not sleeping’ (Mum*, *29*, *of girl 10 months)*
F. Individually focused and non‐generic	Information or advice from sources or individual(s) which was specific to the child, family or individual circumstances and not just general information	*‘All babies have their own personality traits and do not necessarily fit the books image of a typical baby’ (Mum, 29, of girl 10 months)* *‘They actually know my son and do not provide generic advice’ (Mum*, *27*, *of boy 16 months)*
G. Easily and quickly accessible	Sources which were easily and quickly accessible as and when required	*‘…given time constraints they are the most practical also’ (Mum*, *32*, *of boy 10 months)* *‘…it's available 24/7. I do not need to make an appointment’ (Mum*, *30*, *of boy 25 months)*

#### 3.3.1 What did parents prefer or find most useful about resources?

##### Theme A. Experience

For many parents, it was important that information or advice was provided by someone whom they perceived as having pertinent experience. Parents used a range of resources (formal and informal) to access experience‐based information and for many, this represented the best form of understanding:

*‘Experience is the best knowledge’* (Mum, 28, of girl 19 months).

In many cases, parents showed a preference for information or advice that was based on other parents' experiences, primarily because this type of advice was practical and real‐life, to which they could relate:

‘*…advice from other parents who have endured and relate to what I'm going through and express what has worked for them’* (Mum, 28, of boy 29 months).

Those who did prefer using HCPs reported the benefit being in the range of their experience and knowledge:

*‘Health visitors should be aware of all the latest research and be able to advise’ (Mum*, *40*, *of boy 32 months)*.

However, the practical advice that was obtained from other parents was perceived by some parents to be more credible, realistic and acknowledged their own challenges more than that provided by other resources:

*‘It's helpful to speak to other parents who can give a real impression of life with a baby rather than examples in book or online which may not be specific or real’ (Mum*, *28*, *of boy 6 months)*.

What constituted appropriate experience varied between parents with some showing a distinct preference for the personal experiences of other parents. Parents′ preferences for ‘practical advice’ related to both the content of advice but also, crucially, setting that in context with an appreciation of the wider challenges parents may be facing, which went beyond the perceived, limited, scope of some other resources.

##### Theme B. Sources that were in‐line with the parenting approach

Parents most commonly, if not exclusively, selected information from sources which were in‐line with their parenting style:

*‘…mums in this group have similar approach so helps me to feel our sleep is more normal!’* (Mum, 35, of girl 34 months).

Some parents actively sought resources that reflected their desired parenting approach. Specifically, in the current sample, some parents preferred gentle and/or attachment parenting approaches to managing child sleep. Many existing resources were reported to not meet parents' needs due to contradicting their fundamental parenting beliefs. This resulted in some parents reportedly actively avoiding certain resource types, predominantly HCPs, because they believed their advice was not in‐line with their parenting approach:

*‘I wouldn't even bother going to health professionals as my expectation is they would advise you to leave baby or child to cry it out…I don't believe any mainstream resources would support us’* (Mum, 34, of girl 26 months).

Identifying the type of information desired before seeking advice may be specific to parents who adopt gentle or attachment style parenting as, conversely, parents did not commonly report actively seeking behaviourally based methods. Much of the advice and/or treatment methods that would be provided to parents from HCPs, whose advice is based on the strongest evidence base, would be behaviourally based in nature. This type of approach, even though evidence‐based, may contradict and therefore be unacceptable to those with, some specific beliefs about approaches to parenting.

##### Theme C. Support and re‐assurance

In many cases, it was not just the content of the information that was key for parents but also, and in some cases most importantly, the broader support, reassurance, and the ‘human element’ offered:

*‘Doesn't always solve issues but helps you realise everyone has them. Knowing others are in the same/similar boat does wonders’ (Mum*, *37*, *of girl 14 months)*.

For many parents, this type of support predominantly came from sources based on ‘personal experience’ (as highlighted in Theme A). Receiving support and reassurance helped some parents feel more confident in handling their child's sleep:

*‘Talking to other mums reassured me my baby's sleep pattern was common and helped me feel calmer about the way I managed him’* (Mum, 28, of boy 6 months).

Also of importance was that parents perceived that the source, individual or information could be trusted:

*‘The crucial thing here is that I trust these people’ (Mum*, *25*, *of girl 9 months)*.

Other parents were perceived to have the best understanding of the realistic challenges associated with child sleep, and so could understand and support those experiencing similar challenges. Notably, many parents felt that this type of support and reassurance was lacking from HCPs.

##### Theme D. Having access to a broad range of information and the ability to select what is relevant

Whilst some parents showed a preference for accessing particular resources, as described above in Theme B, many indicated it was preferable to have the ability to access a broad range of sources, principally as it allowed them to explore a variety of options and ideas:

*‘We are looking for a balanced opinion on what to try…we've found it most useful to get a variety of ideas before deciding what to try’* (Mum, 35, of boy 6 months).

Some parents actively sought information from different perspectives, so that they could reach an informed decision:

*‘Personally*, *I like to consider different options and then weigh them up*, *what is best for me*, *my baby*, *our family ‐ how will it impact our routines*, *our work/life balance’ (Mum*, *40*, *of boy 24 months)*.

What was key to many parents was being able to review a range of resources and select only the aspects of information or advice that they felt would be useful to them. This information‐seeking approach enabled parents to acknowledge the individuality of their child, their parenting style and their circumstances.

#### 3.3.2 What were parents′ general reservations or barriers to using resources?

The reservations parents held or factors that they perceived to act as barriers when consulting resources for child sleep were also identified. As with their preferences, their reservations also cut across specific types of resources (Table 2).

**TABLE 2:** Parental reservations or barriers to using sources for child sleep

Themes	Description	Example quotations
A. Parental concerns of reliability	Parental concerns about the reliability, evidence base or potential bias of the sources or individuals delivering information or advice	*‘Impossible to know how reliable a source is or whether it is truly evidence based’ (Mum*, *27*, *of girl 19 months)* *‘General information seems to be everywhere. The question is*, *is it evidence based*, *neutral or reliable?’ (Mum*, *34*, *of girl 15 months)*
B. Judgemental	Parental concerns about being generally judged due to their child's sleep issues. There were also concerns that certain sources may be judgemental or critical about their handling of the issues	*‘Personal experience can cloud judgement and people have fixed ideas about how children should sleep and can make you feel judged’ (Mum*, *35*, *of boy 17 months)* ***‘**I do not wish to be judged by friends and family on the fact my little one does not sleep through whereas their children might do’ (Mum*, *23*, *of boy 7 months)* *‘Advice is too emotionally pressured especially for a new/ first time mother’ (Mum*, *45*, *of girl 24 months)*
C. Previous negative experience of source	Previous negative experience of a particular source or source type	*‘Didn't receive very helpful support in the past’ (Mum*, *36*, *of girl 17 months)* *‘I would never Google anything because you have too many opinions to read and I feel you end up more paranoid than when you began’ (Mum*, *30*, *of boy 34 months)*
D. Hard to filter information	Parents found it challenging to identify, obtain or filter information from a specific or multiple sources	*‘Because sometimes I read too much information and have no idea what to try (Mum*, *30*, *of boy 25 months)* *‘So many conflicting views it is sometimes difficult to know what to pay attention to’ (Mum*, *34*, *of boy 12 months)*
E. Information too general	Parental concerns about the relevance of the information. In many cases, information obtained was perceived to be too generic to be useful	*‘Lack of time to really understand issue and therefore giving of generic advice’ (Mum*, *33*, *of girl 9 months)* *‘All babies are different so these sites may offer good general advice but it may not work’ (Mum*, *32*, *of girl 11 months)*
F. Conflicting information or advice received	Parental concerns or confusion due to receiving conflicting or opposing information or advice from sources	*‘…it's insane how many are handing out such conflicting and awful advice’ (Mum*, *34*, *of boy 22 months)* *‘There's a lot of conflicting advice*, *which can be confusing’ (Mum*, *27*, *of boy 11 months)*
G. Limited accessibility	Issues around lack of accessibility, particularly in relation to sources that required parents to attend in person or make appointments	*‘It's very difficult to get appointment with GP or midwives and baby clinics may be at an inconvenient time’ (Mum*, *34*, *of boy 23 months)* *‘One of the barriers to seeking advice from professionals is the difficulty (time and access) in getting to see them’ (Dad*, *34*, *of girl 31 months)*

##### Theme A. Parental concerns about reliability

Parents held concerns about the reliability of the information. One mother highlighted a broad range of concerns about the reliability of information:

*‘Interpretation. Relevance. Fact versus fiction. Old wives' tales’* (Mum, 40, of boy 24 months).

Although this concern was particularly pertinent in relation to online sources, some parents highlighted that they discriminated between online sources based on their perceived reliability as making use of resources that they perceived to be based on reliable information allayed concerns:

*‘I generally prefer to take advice from trusted websites such as the NCT [National Childbirth Trust] or NHS [National Health Service] websites as they contain factual information with references’* (Mum, 29, of girl 16 months).

Interestingly, some parents were concerned about the reliability of advice from HCPs, with many of these parents not perceiving HCPs to possess up‐to‐date, evidence‐based knowledge or that they would deliver reliable advice:

*‘They [HVs] are not kept up to date on research*, *they want “quick fixes”’(Mum*, *29*, *of boy 35 months)*.

However, it was clear that many parents were aware of and concerned about how reliable the information was and that the desire for well‐evidenced information motivated some parents to make use of professional healthcare resources.

##### Theme B. Judgemental

There was an overarching fear for parents of being judged by people who were involved in delivering advice or support across the range of resources. Parents were most concerned about judgement from HCPs, other parents, or family members. One mother reported that interactions with HCPs:

*‘…results in a lot of parents lying to health professionals because they feel like they are being judged’* (Mum, 29, of boy 25 months).

Due to societal expectations and social pressures, child sleep was reported as a topic which could generate competition between parents:

*‘[I] don't listen to friends with similar aged children as it's a lot of competition about whose baby sleeps better’* (Mum, 26, of boy 12 months).

This reservation was most strongly reported in relation to family and friends, but also for online forum‐style sources.

Parents held concerns about potential negative emotional or relationship consequences of using certain sources for information or advice. This was particularly pertinent for relationships with those close to them:

*‘Once you ask for their advice [family]*, *you often are then badgered about whether you have followed it and if we do not agree with it*, *we will not follow it. This is a situation I like to avoid’ (Mum*, *44*, *of girl 21 months)*.

It was clear that sleep was a contentious subject about which many parents were concerned that their problems or approaches would be criticised by others. It was particularly worrying that parents may neglect to discuss sleep‐related issues with HCPs due to concerns about judgement.

##### Theme C. Previous negative experience of source

Previous personal or hearsay of others' negative experience of a resource had a detrimental influence on parents' own perception and use (current and future) of that source:

*‘I would be very cautious about using the Internet for information as this is where I got myself in a right pickle’* (Mum, 36, of girl 17 months).

Online and HCP resources were common sources where the contentious nature of child sleep could be problematic and result in negative experiences:

*[Health Visitor] ‘Never again ‐ I have received nothing but outdated and conflicting advice*, *given without me asking and with plenty of snap judgements’ (Mum*, *34*, *of boy 22 months)*.

It is clear that any negative experience could have a profound impact on how useful resources were perceived to be and how likely parents were to make use of them in the future.

##### Theme D. Hard to filter information

This theme, predominantly but not exclusively highlighted in relation to online resources, was the challenge of finding, obtaining or filtering information:

*‘The internet gives you a lot of results and sifting through them to find things that are relevant is so difficult’* (Mum, 31, of girl 18 months).

For some, the sheer volume of information was overwhelming to understand and synthesise:

*‘I was becoming low and anxious with all the conflicting advice online and from books’* (Mum, 36 of girl 17 months).

It is interesting that the volume of information available or the number of ‘hits’ resulting from a general Internet search was, as previously discussed, viewed as beneficial by some parents, for offering a range of information and options but also disliked and found to be overwhelming by others.

## 4 DISCUSSION

It was clear there were key parental preferences and reservations about child sleep information resources, which aligned with previous findings on other samples of typically developing children of a similar age. Parents desire and value child sleep resources that; (a) reflect other parents' experiences as they deemed them to have the most relevant hands‐on experience (Brady & Guerin, 2010; Hatton & Gardani, 2018; Tsai et al., 2014); (b) were in‐line with their parenting beliefs (Tsai et al., 2014); (c) offered broad emotional support (Bernhardt & Felter, 2004; Drentea & Moren‐Cross, 2005). In addition, parents wanted access to a range of information and options from which they could make informed decisions about what best suited their individual circumstances. Reservations which influenced parents' perception and use of sources included (a) worries about their reliability, particularly for online resources (Porter & Ispa, 2013; Scullard et al., 2010) and (b) concerns about being judged (Hatton & Gardani, 2018). Additional novel findings were that (c) previous use (their own or others') of resources with a non‐favourable outcome was influential and (d) the volume of available information about child sleep felt overwhelming.

The high proportion of parents who reported having sought information or advice for their child's sleep emphasises the importance of the topic (Porter & Ispa, 2013; Trajanovska et al., 2010). Sleep problems in infants and toddlers are common but highly amenable to intervention (Mindell et al., 2006). However, to be appropriately ‘treated’ parents need to seek help. Whilst there is a range of resources available, the current results suggest that there are further adaptations to formal health care or evidence‐based material, which could ensure they best meet parental needs. There are parallels in the challenges experienced by parents in the current study in terms of their sleep‐related help‐seeking and what parents want from sleep resources, as reported in more diverse populations, such as parents of children with epilepsy and autism (Cook et al., 2020; Tan‐MacNeill et al., 2020). This suggests that existing resources (content, format and delivery method) may not be most appropriate for many parents. Perhaps, ensuring sleep resources designed for parents address some of the key themes identified in this study would contribute to ensuring parents' experiences of help‐seeking are beneficial and meet diverse parents' needs at different time points?

There is a clear challenge in synthesising the sometimes‐contradictory issues raised by parents when it appears there is variation in what parents want. For example, many parents reported preferring resources of an informal nature, with some even avoiding formal healthcare resources, even though parental concerns about reliability were widespread and many parents noted that discriminating between vast amounts of conflicting information was problematic. In addition, there was a desire for diverse material but also content which met specific parenting values. Whilst these tensions may appear contradictory, perhaps they reflect different stages or approaches to help‐seeking at different time points. A new sleep intervention developed in the UK for parents of infants has acknowledged the need for broader parenting approaches to be incorporated into child sleep resources (Ball et al., 2020). The key implication of the current findings is that there is a need to fully integrate what parents do and do not want from child sleep resources and address some of the clear tensions identified in the current study in the future development of resources.

General Internet searches were the most used resource by parents and this reflects the trend of parents' increasing use of online resources to seek information about child sleep (Allen & Rainie, 2002; Khoo et al., 2008). Online delivery has been shown to be successful for the delivery of interventions for paediatric sleep (Mindell et al., 2011a; Mindell et al., 2011b) and recent evidence suggests that parents regularly use websites which they perceive to be reliable for child sleep (Simard & Pilon, 2021). Sleep specialists have begun to develop evidence‐based online content delivered in a user‐friendly fashion that includes information, advice and resources about child sleep for parents and HCPs (see Mindell et al., 2021 for an example of babysleep.com. Also available is https://www.basisonline.org.uk/). The use of Babysleep.com has continued to develop (800,000 users from around the world) since launching in October 2016 suggesting it is a well‐needed resource (Mindell et al., 2021). However, although there is evidence of the efficacy of this type of content (Leichman et al., 2020), a formal evaluation of the effectiveness of these websites has not been established.

Parents clearly want evidence from people that they trust and whom they believe will offer them realistic advice and broader emotional support. Whilst many parents appeared to have faith in the reliability of National Health Service (NHS) resources and healthcare services there remained limitations to these, most notably that they are not perceived as providing a broad range of options for parents to review before making their own personal decisions (due to being mandated to provide only evidence‐based information). Therefore, perhaps the most appropriate and realistic solution would be a tiered system focusing on the content and delivery of material. At the lowest level providing a well‐evidenced online resource, which includes information across a range of sleep‐related topics covering different approaches to management which is hosted by a trusted health organisation (in the UK such as the NHS), could be a useful starting point. This may necessitate incorporating information about a range of parenting approaches, including gentle or attachment parenting, and presenting (and regularly updating) the current state of evidence and knowledge so that parents are fully informed. The COVID‐19 pandemic and the increase in reported child sleep problems (Dondi et al., 2021) have emphasised more strongly than ever the need for a good quality centralised online resource that offers support and guidance around infant and toddler sleep, which parents can be directed to. However, individual parents are likely to vary in terms of their health literacy and as such, the usefulness of such resources cannot be expected to act in the same way for all parents. However, a centralised online resource aimed at UK parents appears to be an appropriate first step in supporting parents around their child's sleep.

For some parents, additional help may still be needed to assist them in managing child sleep, including more specialised advice or emotional support. Whilst many HCPs, particularly HVs, are already mandated to offer this type of service alongside evidence‐based advice and guidance, the current study and previous research suggest further adaptations to what these services offer and how it is delivered are required to best meet parents' needs (Cook et al., 2020; Hatton & Gardani, 2018). For example, acknowledging the range of management or intervention options available and providing details of the evidence (or lack of evidence) for these different approaches. Although, given current monetary and workload pressures it may be challenging for HCPs to extend their offering around child sleep in the existing climate (British Medical Association, 2018; Institute of Health Visiting, 2016).

Our results suggest that incorporating relevant ‘experience’, particularly other parents' opinions and shared experiences, is highly desirable. Interestingly, parents of older children with epilepsy have also reported the desire for other parents' experiences to be incorporated into sleep resources (Cook et al., 2020). One possibility could be to explicitly incorporate ‘real‐life’ parental shared experiences, such as through embedding quotations (written or video) or case studies from parents on key topics or experiences. This approach may also help engage parents with the material offered via formal sources (Hatton & Gardani, 2018). Another practical approach could be joint future resource production with parents, healthcare professionals and sleep ‘experts’ collaborating (as highlighted by Hatton & Gardani, 2018). Working collectively has a range of practical benefits (Hickey et al., 2018) and may help to identify the most viable solutions to address the parental preferences and reservations highlighted. Parents and experts have successfully collaborated to identify research questions around key parenting topics, such as feeding (Collins et al., 2020) and to develop an information booklet‐based sleep intervention aimed at parents of infants (Ball et al., 2020). Successful co‐production of an online sleep resource has been achieved for parents of children with epilepsy where several novel amendments (addressing issues such as those reported in the current paper) were made to traditional behavioural sleep intervention delivery to ensure alignment with parental requirements (Wiggs et al., 2021).

These findings need to be considered in the context of some limitations. This was an undefined internet sample which, even though a broad spectrum of parental opinions and experiences were included, is unlikely to be representative of the general population of the UK and may be subject to selection bias due to the nature of data collection. For example, it is clear the current sample comprised participants of above‐average education levels and SES. In addition, there is a lack of ethnic diversity and the current sample does not reflect the general UK population family composition. Future, studies in more diverse populations are needed to further understand cultural and geographic influences on parental help‐seeking behaviours. Nevertheless, a strength is that the current study provides a broad spectrum of parental opinions and experiences and included a thematic analysis of a relatively large sample size of parents. Responders were mainly mothers and so it is possible that fathers or other caregivers may seek help for child sleep in different ways and this requires further exploration.

In addition, as the current data reflect parental beliefs and experiences in 2015–2016, it is possible that since then, and post Covid‐19, parents' use and experiences of resources may differ. However, recent work suggests that existing resources are still not meeting parents' needs (Rhodes et al., 2020; Vazquez‐Vazquez et al., 2021) and that many parents in need of support with child sleep are still not seeking help (Newton et al., 2021). Therefore, there remains a need to understand parents' opinions and experiences of support and resource use in order to understand factors which promote and inhibit parental help‐seeking behaviour and that is provided by the current work.

Finally, questions were developed specifically for use in this study and it is possible that these did not adequately encapsulate all aspects of parents' help‐seeking behaviours. Further exploration using different methods, such as interviews, could be further enlightening.

## 5 CONCLUSION

Current results extended the existing literature by identifying sources used by parents to seek help for infant and toddler sleep, including exploring factors that UK parents preferred and those which created reservations about using sources. Parents made use of a wide range of resources and many of these were commonly informal in nature, with HVs being the only HCP widely used. Parents wanted a range of easily accessible, evidence‐based information provided by someone with relevant experience who offered support and reassurance. They also wanted the freedom to make an informed decision and select only what was deemed to be in‐line with their desired approach to parenting and suitable for their individual circumstances. Many parents did not feel that existing resources adequately met their needs. Future development of resources (materials and services) could helpfully be informed by incorporating parents' views.

### AUTHOR CONTRIBUTION

All authors contributed to the conception, design, analysis, and interpretation of data as well as drafting and critically revising the manuscript for important intellectual content. GC was also responsible for data collection.

## CONFLICT OF INTEREST

The authors declare no commercial or financial conflicts of interest.

## ETHICS STATEMENT

Ethical approval was obtained through Oxford Brookes University Research Ethics Committee (study number 150932).

## Data Availability

The data that support the findings of this study are available on request from the corresponding author. The data are not publicly available due to privacy or ethical restrictions.

## APPENDIX A

Full list from which parents were requested to select the resources they had used when seeking help for child sleep. Parents could select as many options as were relevant to them

[] Health Visitor.

[] Midwife.

[] Doctor.

[] Other healthcare professionals. Please specify…………………..

[] General internet search for relevant information/websites.

[] Trusted parenting / health website with which I am familiar and know to go to directly to seek information. Please specify …………………..

[] Book. Please specify which book (if possible)……………….

[] Leaflet from healthcare professional (e.g doctor, nurse, health visitor, midwife).

[] Family member. Please specify……………………

[] Friend.

[] Other parents from a group (e.g toddler group, online parenting group, NCT). Please specify what group………………….

[] Children's centre.

[] Nursery or childcare facility.

[] Other. Please specify…………………………….
